# Shoulder Pseudo-Tumor from COVID-19 Vaccine

**DOI:** 10.3390/vaccines11040793

**Published:** 2023-04-04

**Authors:** Anas M. Abbas, Timothy A. Damron

**Affiliations:** 1 Norton College of Medicine, SUNY Upstate Medical University, Syracuse, NY 13202, USA; 2 Department of Orthopedic Surgery, SUNY Upstate Medical University, Syracuse, NY 13057, USA

**Keywords:** SARS-CoV-2, education, orthopedics, COVID-19, vaccine, tumor, edema, SIRVA, shoulder

## Abstract

Hypersensitivity reactions to the COVID-19 mRNA vaccines were identified in the initial 2020 trials. Appearance of a soft tissue mass is a rare manifestation of this hypersensitivity reaction. In this patient, bilateral injections resulted in the appearance of shoulder masses. Magnetic resonance imaging showed localized pseudo-tumorous edema in both shoulders, one subcutaneous and the other intramuscular. This is only the second case of a mass-like reaction to the COVID-19 vaccine mimicking a possible soft tissue neoplasm. Improper vaccination administration technique may have contributed to this complication. The case is presented to increase awareness of this potential pseudotumor.

## 1. Introduction

Phase 3 trials of the mRNA-1273 (Moderna) COVID-19 vaccines found delayed injection-site inflammatory reactions known as type IV hypersensitive reactions in 0.8% of participants after the first dose and 0.02% of participants after the second dose [1]. Not exclusive to COVID-19 vaccines, prior vaccines also documented hypodermal reactions. Injection-site granulomatous nodules were reported from the pertussis, anthrax, and botulinum F toxoid, as well as the COVID-19 vaccines [2,3,4]. Granulomas are formed after a persistent inflammatory reaction caused by an inability of macrophages to clear antigens [4]. Aggravation at the injection site was also attributed to vaccine placement during the injection [5]. Depth and needle size may contribute to compensatory inflammation within the subcutaneous or intramuscular tissue planes [5]. Common symptoms that manifest include urticaria, induration, tenderness, and swelling at the injection site. In very rare cases, such as in this patient, the swelling may be mistaken for a neoplasm [6].

Type IV hypersensitivity-related swelling is not the only vaccine related adverse reaction that may come to the attention of the orthopedic surgeon. Shoulder injury related to vaccine administration (SIRVA) is an uncommon vaccine injection complication that causes prolonged shoulder pain [7,8]. It is caused by improper injection into the shoulder and is most commonly presented with associated subacromial bursitis and edema [7,8]. Clinical manifestations of swelling and erythema from SIRVA overlap with typical clinical manifestations of a type IV hypersensitive reaction.

We present a very rare case of the COVID-19 vaccine prompting the growth of an injection site pseudo-tumor. The case is presented to inform proper vaccine injection technique and to bring awareness to this rare clinical occurrence that may mimic soft-tissue tumors.

## 2. Case Presentation

A 53-year-old female presented with an 8-month history of firm, posterior-lateral, bilateral upper-arm soft-tissue masses. The location and temporal onset of the masses were directly related to her first COVID Moderna vaccine injection in her right shoulder and the second Moderna shot 1 month later in the left shoulder by her pharmacist. A discussion with the patient suggested that both vaccines were administered at approximately three finger breadths below the acromion. The patient experienced typical transient shoulder soreness in her right shoulder immediately after the first injection, which was unrelated to the soft tissue masses and associated symptoms. During her second injection, she did not have symptoms in her right shoulder, but she preferred to have the second injection in the left shoulder because she is right-hand dominant. Two months later, she developed bilateral injection-site pain, urticaria, decreased active range of motion, induration, and tenderness. The left shoulder had a less florid reaction than the right. The patient reported her pain as 5 out of 10 on the visual analog scale. The pain was described as a persistent discomfort which limited activities of daily living including driving, cooking, and work. These areas of localized swelling failed to resolve, despite treatment with heating pads and ice packs. No other trauma occurred, there was no history of underlying medical, oncologic, orthopedic, or rheumatologic conditions, and there were no prior known soft-tissue masses. She had no history of drug allergy or allergic comorbidities. There were no constitutional symptoms or adenopathy.

**Figure 1:**
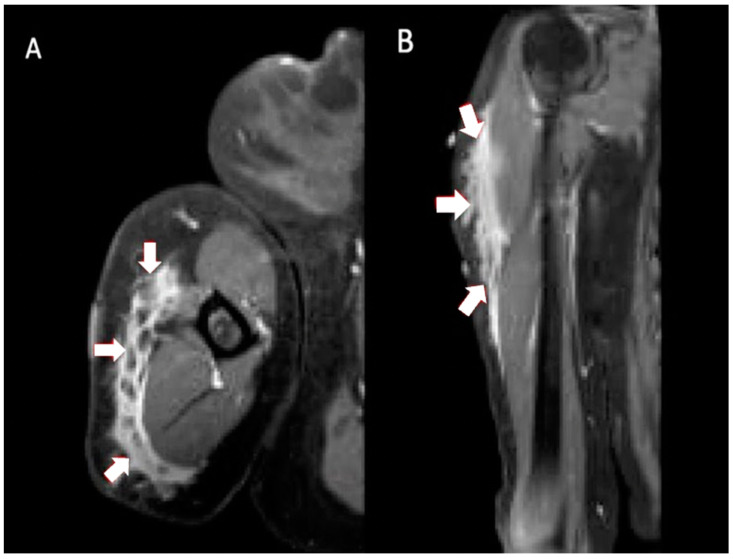
Multiplanar MRI of right shoulder taken 8 months after vaccine administration in right shoulder. Axial T2 (**A**) and coronal T2 (**B**) images show ill-defined fluid signal surrounding interspersed areas of fat. The involved area extended from surgical neck of the humerus to distal diaphysis, measuring approximately 14.9 × 8.2 × 1.8 cm (arrows).

Initially unconcerned of her symptoms, the patient took intermittent Tylenol for the pain and initiated a home exercise program. She had an appointment with her primary care physician (PCP) for an annual check-up 2 months after the symptoms had developed (4 months after completing the injections). Her PCP referred her to orthopedics due to concerns of bilateral shoulder pain and swelling of unknown etiology. She waited another 3 months before consulting orthopedics. By this time, her pain ceased but the bilateral swelling persisted. Her initial treating orthopedic surgeon ordered a right shoulder magnetic resonance imaging (MRI) to rule out malignancy on the more swollen side. The imaging showed abnormal regions within the subcutaneous tissue at the sites of the mass (Figure 1). Subcutaneous edematous tissue extended from the level of the humeral surgical neck to the distal diaphysis. Minor laboratory abnormalities of uncertain significance were noted in bloodwork that were obtained seven months after the second injection (Table 1). Based on the imaging findings in the context of her history of vaccination into the deltoids and improvement in pain over time, her idiopathic reactions were associated with the vaccination, and so, no biopsy was carried out, and observation was elected.

**Figure 2:**
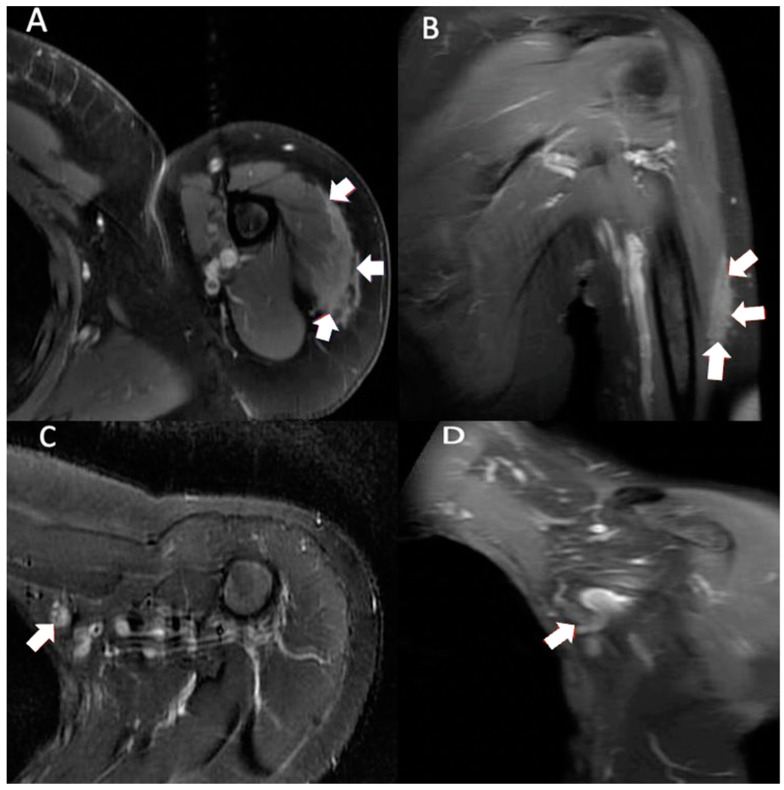
Multiplanar MRI of left shoulder taken 10 months after vaccine administration in left shoulder. Axial T1 weighted (**A**) image shows predominately intramuscular edema within the lateral deltoid muscle measuring 5.6 cm anterior-posterior and 1.0 cm medial-lateral (arrows). Coronal T1 weighted (**B**) image shows intramuscular edema within the lateral deltoid muscle measuring 10.0 cm proximal-distal (arrows). Axial T2 STIR (**C**) and coronal T1 weighted (**D**) images show a slightly enlarged left axillary node (9.9 × 11.2 × 12.8 mm) (arrows).

Three months after visiting the initial orthopedist, the patient attended a different orthopedic clinic to address the masses. At this visit, 10 months had passed since receiving her injections and the clinical examination showed normal range of motion with no tenderness to palpation. There were no rashes on inspection; however, edema and nodules to palpation were found in the lateral deltoid heads. Due to the persistent, previously unimaged left shoulder swelling, an MRI was performed, which showed similar findings of edema but in an intramuscular rather than subcutaneous location and the presence of a slightly enlarged axillary node (Figure 2). Ultimately, no biopsy or treatment was recommended on either side given the imaging findings and the improvement, albeit slow, in her symptoms and the swelling bilaterally. The patient’s symptoms had fully resolved when she returned after 2 months (1 year after completing the injections). Examination showed 5/5 deltoid strength bilaterally, full active range of motion without discomfort or crepitus, no visual deformities (swelling, effusion, erythema) in the distal upper extremities, and no joint tenderness on palpation.

She did not experience similar reactions to prior vaccines. The adverse reactions to the initial vaccine injections dissuaded the patient from receiving a recommended booster dose for COVID-19.

## 3. Discussion

Type IV hypersensitivity reactions following COVID-19 vaccination are known as “COVID arm” [9]. They were reported almost exclusively in association with mRNA vaccines [9]. These are delayed, localized reactions with a median onset of symptoms within one week and median duration of five days following the first dose of the Moderna vaccine [6]. Delayed reactions following the second Moderna shot were milder with a shorter median onset and duration (median onset = 2 days, median duration = 3 days) [6]. A case series found similar results in the timing of urticarial reactions in the first and second doses between the Moderna, Pfizer, and AstraZeneca vaccines [10]. However, this study found a lower recurrent rate of adverse events following the second dose; four of eighteen patients experienced a subsequent urticarial reaction [10]. Typical clinical manifestations included erythema, induration, urticaria, and tenderness [6,10,11]. Histologic examinations from three separate case series found local perivascular mixed infiltrate of predominantly CD3+ T cells and eosinophils in symptomatic patients [6,11]. Our patient received an injection into each shoulder as opposed to the patients in the case series that received both injections in the ipsilateral shoulder [6,10,11]. Pre-existing lymphocytic or immune cell infiltrates from prior injections would decrease the duration of symptoms in subsequent reactions. In our case, however, one injection into each shoulder excluded this possibility, leading to prolonged duration of pain, urticaria, and induration. No biopsy was performed in this case, which might have shown the granulomatous nature of the reaction since the patient’s imaging and clinical course did not justify it. The type IV immune reaction was associated with persistent indigestible antigens that prolong the inflammatory response. This incidental inflammation results in the granulomas of COVID arm, but it does not usually result in the pseudo-tumorous swelling seen in this patient. In this case, we hypothesized that the patient experienced a florid type IV hypersensitivity reaction leading to a granulomatous response in a nodular and edematous fashion.

Specific vaccine components that elicit a granulomatous reaction were identified. In the pertussis vaccine, aluminum salt adjuvants were identified as the responsible ingredient that led to a reaction in 77% of children [3]. While the Moderna vaccine does not contain this ingredient, polyethylene glycol (PEG) and tromethamine are two ingredients found in the Moderna vaccine which were implicated in injection reactions [6,12]. Tromethamine was linked to anaphylactic reactions, but there were no studies associating it with hypersensitive reactions [12]. PEG inoculation in a rabbit aorta model resulted in intense granulomatous reactions [13]. However, the effects of PEG in delayed reactions were not reported. The patient’s abnormal leukocyte counts may have been related to the delayed inflammation at the injection sites, resulting in an increase in monocytes and neutrophils consistent with the inflammatory cascade. Monocytes initiate the granulomatous reaction by recognizing foreign antigens, possibly PEG or tromethamine, and activate neutrophils which release cytokines and increase capillary permeability to recruit immune cells, exacerbating the localized granulomas. The prolonged symptoms of this patient conveyed an inability of leukocytes to identify and clear foreign substances. Anatomic variation in deltoid vasculature is a possible explanation of inoculation into regions with poor blood supply, thus impeding immune cell infiltration. Further studies need to be carried out to identify specific mRNA vaccine components that may contribute to type IV hypersensitivity reactions. Although our patient had no drug allergies, it is unlikely that she was tested for allergy to PEG or tromethamine. The patient had not experienced similar reactions to prior vaccines. Assuming that her prior vaccines were administered with a similar technique, the inoculation of certain ingredients such as PEG or tromethamine may have led to the localized reactions.

With respect to treatment, a type IV hypersensitivity granulomatous reaction, as presumed to have occurred in this case, typically resolves without medical intervention, possibly led by an in vivo reactive oxygen species-mediated cascade [14].

Injection site granulomas were attributed to improper injection technique [15]. The intended location for COVID vaccination is intramuscular (IM), usually into the bulk of the deltoid muscle [5]. Injection site granulomas tend to occur in subcutaneous tissue (SC) and more frequently in females [15]. The depth of vaccine injections has a strong impact on tissue reaction. Deep IM injections are typically associated with fewer symptoms compared to SC or intradermal injections, possibly due to lower levels of nociceptors in muscle spindles compared with subcutaneous tissue and skin [16]. This likely accounts for the lower amount of swelling and pain on the patient’s left shoulder, where the imaging showed the reaction to be intramuscular, in contrast to the more symptomatic right shoulder subcutaneous reaction.

Other than granulomatous hypersensitivity reaction, a neoplasm and SIRVA were also considered in the differential diagnosis for this patient. In any presentation of an unexplained soft tissue mass or localized swelling, a neoplasm must be considered in the differential. Arguing against that in this case, however, were the bilaterality, temporal association with vaccination, and relatively diffuse nature of the process on each side. Ultimately, imaging excluded this possibility, and so, no biopsy was performed. Neoplastic etiology was unlikely, and we believed that this inflammatory reaction led to the appearance of pseudo-tumors that could be mistaken for a neoplasm.

SIRVA is attributed to incorrect injection technique with injection into the shoulder joint, rotator cuff, subacromial bursa, or regional peripheral nerves [7,8]. The diagnostic criteria for SIRVA are absence of shoulder pain prior to vaccination, rapid onset of localized pain, and limited range of motion in the vaccinated shoulder within 48 h [7,8]. The most common results are subacromial bursitis and edema [7,8]. Although an unusual presentation of SIRVA was considered in this patient, the more predominant presentation with localized swelling accompanied by urticaria rather than severe pain was more consistent with a hypersensitivity reaction [6]. Patients with SIRVA had prolonged pain which were treated commonly with corticosteroid injections (subacromial bursitis) or surgery (nerve injury) [7,8].

To prevent these rare complications, proper injection techniques should be used. The deltoid is the most common IM injection site due to easy exposure of the overlying skin [5]. Deltoid IM injection is a safe zone for vaccine administration, but deep needle placement may affect other unintended targets that may be associated with SIRVA [5]. A proper injection technique is described as insertion of a needle at a 90° angle with a depth 5 mm greater than the subcutaneous thickness at 1–3 finger breadths (5 cm) below the mid-acromion [5]. The vaccinee should place their hand on the ipsilateral hip and abduct the preferred arm at a 60° angle [17]. According to the patient, a proper landmark was found in the bulk of the deltoid muscle, based on her description of the injection site below the acromion. The subcutaneous edema found in the MRI of right shoulder lead us to believe that the needle length used, or the angle of administration, resulted in the needle tip not reaching the intramuscular tissue plane.

## 4. Conclusions

Adverse reactions to vaccines are usually mild and transient. Unfortunately, several cases of prolonged symptoms were reported, possibly linked to improper injection technique. A neoplastic condition should be considered in the differential diagnosis when swelling occurs post-vaccination, but imaging distinguishes this inflammatory response from a potential tumor. The information presented in this case may be used to inform providers on proper injection techniques to decrease the incidence of vaccine complications. In addition, orthopedic oncologists should be aware of pseudo-tumors following vaccination before ordering biopsies. While SIRVA or type IV hypersensitive reactions are possible, their uncommon occurrence should not deter patients from obtaining the recommended vaccinations for COVID-19. An initial reaction is not a contraindication to subsequent boosters.

## Figures and Tables

**Table 1:** Complete blood count with differential taken 7 months after completing primary series of vaccination.

Lab	Profile	Range	Units
RBC	4.24	4.00–5.40	10.0
Hgb	12.3	12.0–15.5	g/dL
HCT	38.3	36.0–47.0	%
MCV	90.3	80.0–96.0	fL
MCH	29.0	27.0–33.0	pg
MCHC	32.1	32.0–36.5	g/dL
RDW	13.2	11.5–14.5	%
PLT	320	150–450	10.0
* Neut	69.7	36.0–66.0	%
* Lym	16.3	24.0–44.0	%
* Mono	10.6	2.0–8.0	%
Eos	2.2	0.0–3.0	%
Baso	0.9	0.0–1.0	%
IG	0.3	0.0–3.0	%
NRBCs	0.0	0.0–0.0	%
Neut	4.5	1.5–8.5	10.0
* Lym	1.0	1.5–5.0	10.0
Mono	0.7	0.0–0.8	10.0
Eos	0.1	0.0–0.5	10.0
Baso	0.1	0.0–0.2	10.0
ESR	21	0–30	mm/h
* CRP	13.7	3.0–10.0	mg/L

*, Abnormal values; WBC, white blood count; RBC, red blood count; Hgb, hemoglobin; HCT, hematocrit; MCV, mean corpuscular volume; MCH, mean corpuscular hemoglobin; MCHC, mean corpuscular hemoglobin concentration; RDW, red cell distribution width; PLT, platelet count; Neut, neutrophils; Lym, lymphocytes; Mono, monocytes; Eos, eosinophils; Baso, basophils; IG, immature granulocyte; NRBCs, nucleated red blood cell; ERS, erythrocyte sedimentation rate; CRP, c-reactive Protein; g/dL, grams/deciliter; fL, femtoliters; pg, picograms; mm/h, millimeters/hour; mg/L, milligrams/liter.

## Data Availability

All relevant data are provided in the paper.
